# Changes in Shape, Texture and Airflow Improve Efficiency of Monitoring Traps for *Tribolium castaneum* (Coleoptera: Tenebrionidae)

**DOI:** 10.3390/insects11110778

**Published:** 2020-11-10

**Authors:** Panamulla A. H. Sajeewani, Dissanayaka M. S. K. Dissanayaka, Leanage K. W. Wijayaratne, Charles S. Burks

**Affiliations:** 1 Department of Plant Sciences, Faculty of Agriculture, Rajarata University of Sri Lanka, Puliyankulama, Anuradhapura 50000, Sri Lanka; hasajeewani@gmail.com (P.A.H.S.); dissanayaka.randeniya@gmail.com (D.M.S.K.D.); 2 Agricultural Research Service, USDA, San Joaquin Valley Agricultural Sciences Center, 9611 South Riverbend Avenue, Parlier, CA 93648, USA; charles.burks@usda.gov

**Keywords:** *Tribolium castaneum*, fan, pheromone trap, trap designs, kairomone

## Abstract

**Simple Summary:**

The commercially-available traps for the red flour beetle generally have low trapping efficiency, limiting their effectiveness for accurate monitoring of the population density of this cosmopolitan pest in different storage and food processing facilities. Five new traps were developed, and their efficiency for trapping the red flour beetle was tested. These new traps have the option of operating with or without a fan. The new traps generally had higher trapping than the present commercial trap. Further exploration for use in different stored-product facilities would enhance the use of these traps while providing a better estimate of the population present.

**Abstract:**

The red flour beetle, *Tribolium castaneum*, is an important pest of stored products. We compared an existing standard commercial trap with five experimental trap designs differing from the status quo in shape, surface texture, and in forced air capability provided by fans. We tested the five new traps and a commercial trap with *T. castaneum* adults with the presence/absence of air flow and the availability of either the pheromone only or both the pheromone and kairomone. Without using the fans and baited with pheromone only, these new trap designs capture beetles three to five times as efficiently as the status quo trap. Use of both pheromone and kairomone doubled the capture efficiency of the status quo trap but did not significantly affect the capture efficiency of the new trap designs, all of which captured significantly more effectively than the status quo trap. Turning on fans for forced ventilation significantly improved trap efficiency of the more effective of the newer traps compared to monitoring with both pheromone and kairomone but no fan. This study provides new insights into factors affecting trap efficiency for monitoring of *T. castaneum* in grain storage facilities, and suggests ways in which existing traps might be improved.

## 1. Introduction

Globally, insects cause extensive damage to seed and their various processed food products during storage [1,2,3,4]. *Tribolium castaneum* (Herbst) (Coleoptera: Tenebrionidae) red flour beetle, is a major cosmopolitan pest associated with stored-products, and found in feed mills, flour mills, warehouses, bakeries [5,6,7], processing plants [8], granaries and other farm storage facilities, grain elevators, stores and residences [9]. Its larvae and adults cause damage to wide array of food; including grains, spices, packaged foods [10,11,12], dried fruits and nuts [13,14]. 

Monitoring is an integral part of stored-product pest management [15,16,17]. Traps are important for monitoring the presence/absence as well as determining the distribution of insects [18,19,20]. A wide array of traps have been developed to monitor *T. castaneum* [21,22,23]. The pheromone and kairomone-baited traps available only catch a fraction of *T. castaneum* [24,25,26] suggesting the need for improvements.

Factors previously shown to influence trap efficiency include colour [27], pheromone dispenser, trap placement [28], sound [29,30], temperature, relative humidity, air flow, food spillage accumulation and light intensity [31]. However, studies on the response of insects to trap shape are rare [32]. Food ingredients have also been used in monitoring of stored-product insects either in isolation [7,33] or in combination with pheromones [24].

The aggregation pheromone 4,8-dimethyldecanal (4,8 DMD) is produced by adult males of *T. castaneum*, and attracts both sexes [34,35]. The dome pitfall trap is available commercially and widely used trap to sample for *T. castaneum* [16,21,36,37]. This commercial trap has the *T. castaneum* aggregation pheromone and a food-based kairomone [26]. This food-based kairomone is shown to increase the trapping efficiency beyond the pheromone [33]. Despite its wide usage, there have been certain shortfalls associated with the dome traps. The efficiency of trapping *T. castaneum* insects by this trap is low [37]. Previous field studies suggest poor correlation between *T. castaneum* population distribution and capture in dome traps [31]. Moreover, the trapping of *T. castaneum* by the traps deployed under laboratory conditions is also reported to be low [37] necessitating its improvement. Previous studies have also investigated the possibility to increase the trapping efficiency of *T. castaneum* adults by using commercial dome trap. Certain botanical oils used as kairomone increase *T. castaneum* trapping percentage [33], but further improvement is needed. Recent studies that investigated the possibility of increasing *T. castaneum* trapping percentage in dome traps include tests of different concentrations of pheromone and kairomones at different distances [38], and pre-exposure effect of biorational insecticides such as spinosad on trapping efficiency [39]. Development new trapping devices for *T. castaneum* is another possible avenue for improving monitoring for this serious stored-product insect pest species. 

The dome traps use natural diffusion of air. Previous studies have shown that exhaust air flow downstream of both the pheromone and the adult-releasing-point significantly increases the trapping efficiency of *T. castaneum* adults [26,37]. The objective of our research was to separately test the effects of trap design and forced ventilation. We tested the efficacy of the new traps with *T. castaneum* compared to the unventilated commercially available dome trap with pheromone alone or pheromone in combination with kairomone. 

## 2. Materials and Methods 

### 2.1. Insects

*Tribolium castaneum* beetles used in the experiments were originally collected from a rice mill in Thambuttegama, Sri Lanka and reared in the laboratory since 2014. To obtain the adults required for the experiment, 200 parent adults were reared on 250 g of a rearing medium consisting of wheat flour, dog food and broiler feed in a ratio of 2:1:1 [40] and maintained at 30 ± 1 °C and 65 ± 1% relative humidity inside an incubator (FH-1200 LED T8, HiPoint Laboratory, Taiwan) for 7 days and then removed. Newly-emerged adults from these cultures were used in the experiments one month following their emergence. The adults for a given replicate were randomly selected from these cultures, counted into a vial using a vacuum pump (Rocker 300, Rocker Scientific Co. Ltd., New Taipei City, Taiwan), and used in the experiment.

### 2.2. Commercial Trap

A commercially available pitfall-style dome trap (Storgard The Dome^TM^ Trap, Trece Inc., Adair, OK, USA) was used as a baseline comparison in this study. This dome trap consists of two plastic cups. Inside top cover of the trap has three slots for the placement of pheromone lures. The bottom cup bears a circular reservoir at the centre to place an absorbent paper pad for which food oil is applied and acts as kairomone. The inside of the circular reservoir is smooth to reduce the ability of the insects to escape [41].

### 2.3. Development of New Traps

All the materials used for the fabrication of the new traps were durable materials that were easily and economically obtained at the local market at a low price (Table 1). Three triangular traps, a square-shaped trap and a hexagonal trap with an exhaust fan driven by a motor at the centre were prepared using white colour abrasive papers. A small motor, 1.5 V battery, white colour sand/abrasive papers, exhaust fan (wind speed = 0.8 ms^−1^), electric wires, plastic vials, nylon netting material (each hole 2 mm × 2 mm), rigifoam and small plastic funnel were also used to fabricate the system.

**Figure 1:**
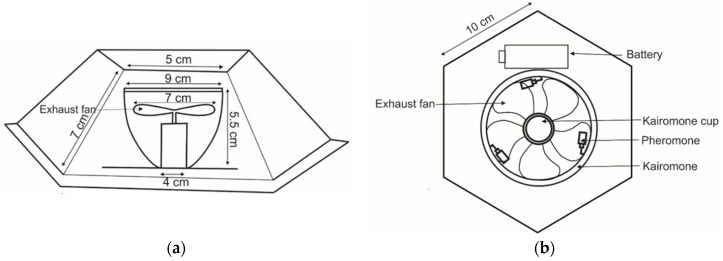
Schematic diagrams of Trap 1. (**a**) Cross section of trap 1; (**b**) Top view of trap 1.

**Figure 2:**
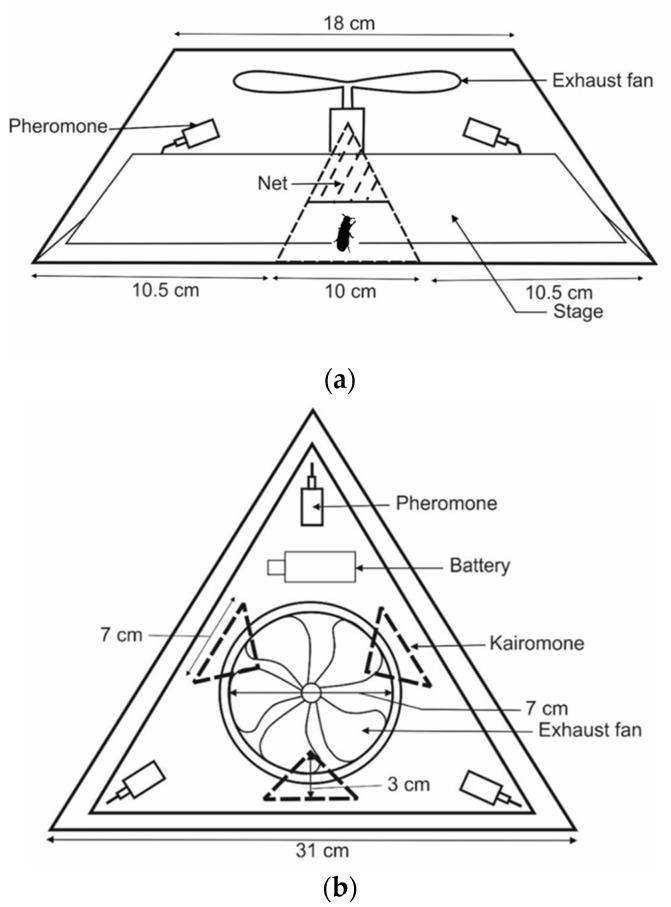
Schematic diagrams of Trap 2. (**a**) Cross section of trap 2; (**b**) Top view of trap 2.

**Figure 3:**
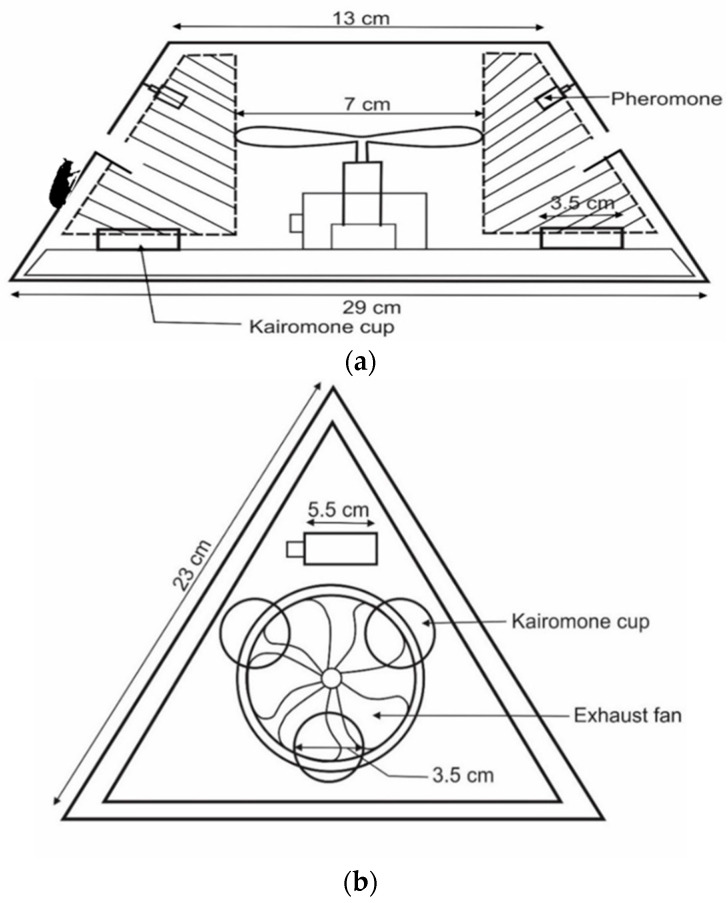
Schematic diagrams of Trap 3. (**a**) Cross section of trap 3; (**b**) Top view of trap.

**Figure 4:**
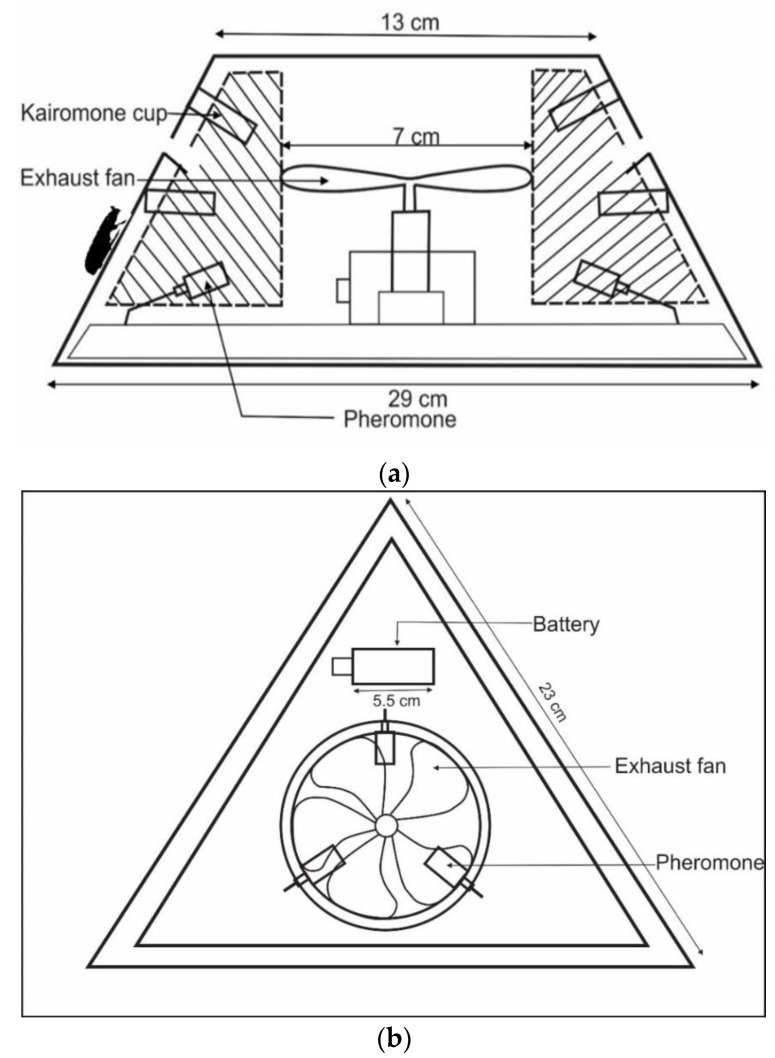
Schematic diagrams of Trap 4. (**a**) Cross section of trap 4; (**b**) Top view of trap 4.

**Figure 5:**
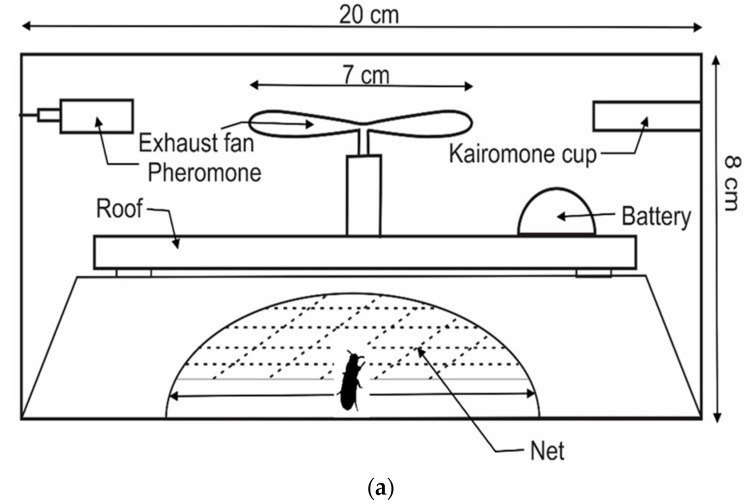

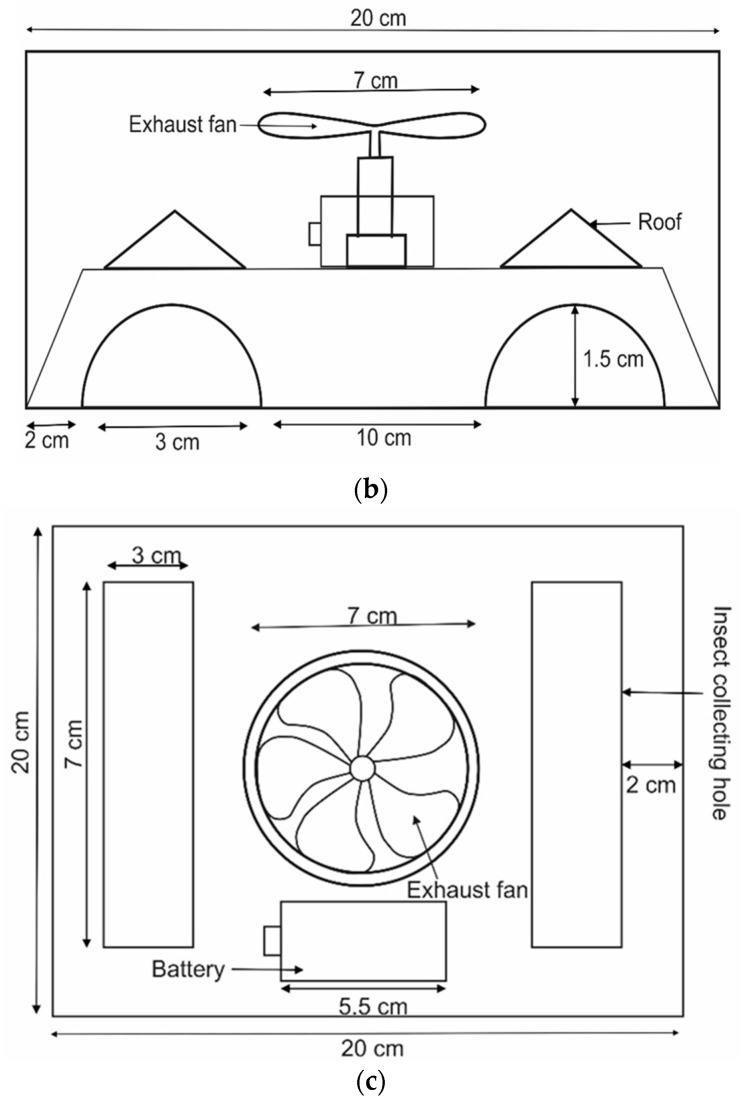
Schematic diagrams of Trap 5. (**a**) Cross section of trap 5; (**b**) Cross section of trap 5; (**c**) Top view of trap 5.

Traps designs varied in shape: Trap 1 (Figure 1 and Figure S1) hexagonal; Trap 2 (Figure 2 and Figure S2), Trap 3 (Figure 3 and Figure S3) and Trap 4 (Figure 4 and Figure S4) triangular; Trap 5 (Figure 5 and Figure S5) square shape. Trap designs 1, 3, 4 were developed using a fine grit sandpaper (grit P 180, Kevin Portugal) to facilitate upward movement of insects into the trap through the openings. In contrast, the trap designs 2 and 5 facilitate entry of insects closer to the ground and were made with a coarser grit sandpaper (grit P 40, Kevin Portugal). Furthermore, in the trap designs 2 and 5, a rigifoam pad (thickness 2.5 cm) (RPC Polymers Pvt Ltd., Maharagama, Sri Lanka) was placed on the bottom of the trap. The coarse nature of this material was expected to facilitate the movement of insects inside the trap.

### 2.4. Pheromone and Kairomone

The aggregation pheromone 4,8-dimethyldecanal (Trece Inc., Adair, OK, USA) impregnated in rubber septa was used. Three rubber septa were used in each trap (new traps or the dome trap). For each experiment, fresh pheromone-containing septa were used. An oil-based kairomone food attractant (Storgard Oil) (Trece Inc., Adair, OK, USA) was added to the filter paper pads and placed inside the reservoir of bottom plate of the dome trap. Similarly, the absorbent filter paper pads were placed inside the kairomone cups in each new trap. From the kairomone, 15 drops were added for each experiment.

### 2.5. Experimental Design

Treatments were arranged as a completely randomized design (CRD). Using the five new trap designs and the commercial trap, the trapping percentage of *T. castaneum* adults under the following categories was determined without and with the operation of fan. Each trap with fan status was tested using the trap baited with pheromone, or with both pheromone and kairomone inside the trap. There were four replicates for each treatment combination. To compare and contrast the trapping efficiencies of the newly-developed traps over the control, the *T. castaneum* adults trapped in the commercially available trap in its natural form was used. All the experiments were conducted between 9 am–1 pm. The temperature, relative humidity and the light intensity during the experiment were 30.2 ± 0.9 °C, 65.2 ± 0.7% and 90.1 ± 0.5 lux, respectively. The temperature and RH profiles during the experiment were measured at every 15 min by using data loggers (TM-305U, Onset Computer Corporation, Bourne, MA, USA). The light intensity was measured by lux meter. The floor of the room used for the study was washed using a biodegradable disinfectant (Britol Disinfectant Pine, Antler Industries Pvt. Ltd., Piliyandala, Sri Lanka). Following each experiment, the room was fully opened to be ventilated to remove any volatile residues from the previous experiment.

### 2.6. Experiments

**Figure 6:**
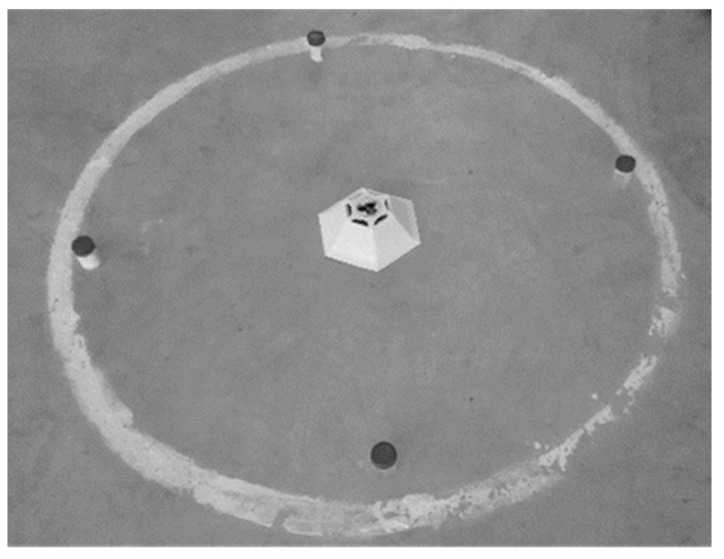
Experimental arena showing the trap in the middle and the circle having a radius of 60 cm on which the beetles were released. Beetle released points are marked in black.

All the experiments for a given replicate (using dome trap and the new traps) were set up and conducted simultaneously (same day between 9 am and 1 pm) at the same location [42]. The different (four) replicates were run on consecutive days. Each experiment was conducted in a separate empty room (8 m × 5 m × 3 m) with cement floor. The rooms used were adjacent and identical, and a single trap was tested in each room on a given day. The ambient environmental conditions during the experiments were 30.2 ± 0.9 °C, 65.2 ± 0.7% r.h. and 90.1 ± 0.5 lux, respectively. A circle with a radius of 60 cm was marked on the cement floor of the room and the new trap was placed at the centre of the circle (Figure 6). This distance was selected based on the findings of previous research that the maximum trap catch for *T. castaneum* is at 60 cm from the pheromone 4,8 DMD [26]. Three rubber septa were placed inside each trap at the designated locations as shown in Figure 1, Figure 2, Figure 3, Figure 4 and Figure 5. Wind power created by the fan inside the trap was measured at 2 cm above the experimental arena by an environmental meter (Brannan, 5-in-1 Multi Enviro-Meter, England). The four directions North, South, East and West were marked on the circle and 50 one-month-old adults of *T. castaneum* were released from each direction separately [25,43]. The experiment was repeated by replacing the new traps with the commercially available trap. Three hours following the introduction of adults, the trapping percentage of individuals trapped in each trap with and without the fan was determined.

### 2.7. Data Analysis

Number of insects trapped in each condition was analysed using Generalized Linear Model of ANOVA using Statistical Analysis System (SAS) [44]. Mean separation was done by Tukey’s test with significance of *p* < 0.05.

## 3. Results

### 3.1. Trapping Efficiency without the Fan

Without the operation of the fan, percentage of *T. castaneum* adults trapped varied with both the trap design (F = 163.44; df = 5,41; *p* < 0.0001) and its lures (either pheromone alone or both pheromone and kairomone) (F = 11.35; df = 1,41; *p* = 0.0016). 

#### 3.1.1. Trapping Efficiency with Pheromone Alone

All the new trap designs had higher trapping percentages than the commercially available trap. Trapping of insects in Trap1 (34.4 ± 0.9%) and Trap 2 (33.8 ± 2.3%) did not differ from each other. The trapping percentages in Trap 3 (60.0 ± 1.4%), Trap 4 (49.8 ± 1.0%) and Trap 5 (42.0 ± 0.4%) significantly differed from Traps 1 and 2. Furthermore, the trapping percentages differed among Trap 3, 4 and 5. Trap 3, the triangular trap with pheromone above and the kairomone below, had the highest trapping percentage (60.0 ± 1.4%), and percent captures were more than three times higher than those observed in the commercially available trap (12.6 ± 1.9%) (Table 2).

#### 3.1.2. Trapping Efficiency with Both Pheromone and Kairomone

All the trap designs had higher trapping percentages than the commercially available trap. Trapping of insects in trap designs 1 (36.0 ± 0.91%) and Trap 2 (33.9 ± 1.9%) did not differ from each other. The trapping percentages in Traps 3 (65.8 ± 1.9%), 4 (50.5 ± 0.7%) and 5 (42.5 ± 1.3%) significantly differed from Traps 1and 2. Furthermore, the trapping percentages differed among Trap 3, 4 and 5. Trap 3, the triangular trap with pheromone above and the kairomone below, had the highest trapping percentage, and was approximately 2.5 times than that of the commercially available trap (23.6 ± 0.6%) (Table 2).

#### 3.1.3. Pheromone Alone vs. Both Pheromone and Kairomone

Only the commercially available trap demonstrated difference in trap catch (more adults were captured in the commercial trap when both the pheromone and kairomone lures were used together compared to the pheromone only treatment) (F = 30.02; df = 1,6; *p* = 0.0015). Trap catches of all the newly-developed five traps were not significantly different between the two conditions (pheromone only vs. both pheromone and kairomone). However, the maximum trap catch by the commercially available trap (with both pheromone and kairomone) was lower than all the trap catch values of newly-developed traps (either pheromone alone or pheromone + kairomone) suggesting that despite the component inside (either pheromone only vs. pheromone + kairomone), all the five new traps caught more insects than the commercially available trap.

### 3.2. Trapping Efficiency with the Fan

As with experiments without the fan, trapping efficiencies also differed among trap types (F = 272.38; df = 5,41; *p* < 0.0001) and between attractants (either pheromone alone or both pheromone and kairomone) (F = 26.38; df = 1,41; *p* < 0.0001).

#### 3.2.1. Trapping Efficiency with Pheromone Alone

The trapping percentages differed between all traps tested (Table 2), and all the new trap designs had higher trapping percentages than the commercially available trap (operated without fan) (F = 148.48; df = 4,15; *p* < 0.0001). Trap 3, the triangular trap with pheromone above and the kairomone below, had the highest trapping percentage (75.0 ± 1.3%), and was approximately five times than that of the commercially available trap (12.6 ± 1.9%) (Table 2).

#### 3.2.2. Trapping Efficiency with Pheromone and Kairomone

Trapping percentages differed in different trap designs when both pheromone + kairomone were kept inside the traps (F = 97.69; df = 4,15; *p* < 0.0001). The percentage beetles trapped varied as Trap 3 (85.1 ± 1.5%) > Trap 5 (75.0 ± 1.7%) > Trap 4 (59.1 ± 1.3%) > Trap 2 (49.3 ± 1.5%) > Trap 1 (37.0 ± 3.1%). Furthermore, all new trap designs had significantly higher trapping percentages than that of the commercially available trap (operated without fan) (23.6 ± 0.6%) (F = 164.54; df = 5,18; *p* < 0.0001). Trap 3, the triangular trap with pheromone above and the kairomone below, had the highest trapping percentage (85.1 ± 1.5%), and was approximately 3.5 times than that of the commercially available trap (23.6 ± 0.6%) (Table 2).

#### 3.2.3. Pheromone Alone vs. Pheromone and Kairomone 

Significant differences in the trap catches between pheromone alone and pheromone + kairomone were observed in Trap 5 (F = 162.31; df = 1,6; *p* < 0.0001) and Trap 3 (F = 150.94; df = 1,6; *p* < 0.0001). Thus, these two trap designs caught higher *T. castaneum* adults when both pheromone and kairomone were used than the pheromone alone. 

### 3.3. Trapping Efficiency with and without the Fan in Operation

There was a significant difference in the trap efficiency with the operation of fan when either the pheromone alone (F = 72.86; df = 1,34; *p* < 0.0001) or both pheromone and kairomones were used (F = 52.29; df = 1,34; *p* < 0.0001). In both cases (either with pheromone alone or both pheromone and kairomones) only the trap designs 2, 3, 4 and 5 had significantly higher trapping with the operation of fan. In contrast, the trapping efficiency of trap design 1 was not improved due to the operation of fan when it had pheromone alone or pheromone and kairomones (Table 2).

### 3.4. Differences in Trapping T. castaneum among Traps

Considering the volatility of pheromone and kairomones used in the traps, it is likely that the number of openings and their placement would have greatly affected the release rate and pattern of release of these two compounds from inside the trap to the outside causing different trapping efficiencies. 

The Trap 3, the triangular trap with “pheromone above and kairomone below” the fan position recorded the highest trapping percentage. This triangular trap has 2 openings on each side covered by netting material (thus, 6 openings in total) and are basically meant for the release of pheromone and kairomones outside. These volatiles could also occur through the horizontal bare opening on each side intended for the movement of insects into the trap. Since Trap 3 is closed on top, there is presumably greater release of pheromone and kairomone out the side of the trap. Thus, a greater proportion of pheromone and kairomone are available around the trap and reach the insects on the floor. The pheromone placed above kairomone would have created a specific movement pattern or/and mixing of two types of molecules ensuring the maximum attraction of beetles to the trap. When the fan is in operation, upward movement of the pheromone may be increased but release may be impeded by the closure of top cover. This would have facilitated much of the pheromone reaching surface of the floor. As the kairomones cups are placed below the fan, the release rate of kairomones might have been less affected ensuring their slow release out. We therefore hypothesize much of the pheromone and kairomone released outside can expected to reach *T. castaneum* adults available on the surface where the trap is placed. However, further experiments using distance-specific mark-release-recapture or quantitative estimates of semiochemical concentration are necessary to test this hypothesis.

The Trap 5 has the pheromone and kairomone at the same level (also with the fan). The release of these two compounds may mostly occur through two large openings to have a higher trapping efficiency. However, the number of openings for the release of pheromone and kairomones are lower than that for the Trap 3. Furthermore, the placement of pheromone and kairomone separately close to an opening would have limited the immediate mixing and/or simultaneous action of these two types of volatiles and thus keeping the trap efficiency lower than the Trap 3 and 4 under no fan effect. However, with the fan effect, the above limitation would have been removed. Accordingly, the trapping efficiency would have been improved than the trap 4.

The Trap 3 and Trap 4 are identical in structure except the difference in the placement of pheromone and kairomone. Thus, the difference in trapping percentages by two traps is likely to be attributed to this relative placement of pheromone and kairomone. The opposite placements of pheromone and kairomones in Trap 4 compared to Trap 3 would have negatively affected the movement pattern of two volatiles. This would have affected their interaction or/and level of perception by the insects and thus reducing its trapping efficiency of Trap 4 lower than the Trap 3. 

The Traps 1 and 2 had lower trapping efficiency than the traps 3, 4 and 5. In the trap 1, higher number of openings (9) and their placement in the vertex would have increased the release of pheromone and kairomones from inside the trap to the outside and subsequent upward movement. This would have caused much of the pheromone and kairomones to be removed from the area where the trap was located. The reduced pheromone and kairomones reached the beetles on the ground surface would have reduced their attraction to the traps. In Trap 2, both the release of volatiles and entry of the insects were facilitated through the same opening. The low number of openings would have reduced the release of pheromone/kairomone and consequently the attraction of beetles to the trap.

Beside those operational differences due to the placement of pheromone and kairomone, the structural differences between new traps and commercial dome traps might also be factors in the different trapping levels. These factors include:

i. The newly developed traps are triangular, square shaped or hexagonal in shape in contrast to the circular-shaped commercially available dome trap. The shape of the trap affects dispersal of the pheromone (+kairomone) from inside the trap to outside and subsequently the rate of pheromone diffusion.
ii. The new traps were developed using abrasive type papers. The commercial trap is made of plastic material. This difference in the surface material on which insect moves would have assisted the stability of its movement into the new traps than the commercial trap.
iii. In the commercial traps, the plane of the surface through which the insects move in is curved (not straight) in contrast to the straight surfaces in the new traps. This aids the insect’s stability on the surface during its orientation towards the pheromone/kairomone inside the trap.

While the above differences in the new traps enhances the trapping efficiency, it will be important to pay attention on the commercialization of them as well. The low cost for preparing one trap (US$ 1.31), small size and their different shapes fitting into different places in food processing facilities make them ideal trapping devices. Despite these advantages, the efficiencies of new traps need to be tested in a food processing/warehouse facility where machineries and other conditions occurred naturally are available.

## 4. Discussion

Air movement is a critical factor in attracting beetles towards the source [45], and the efficiency of trapping *T. castaneum* increases with the presence of air flow in pheromone and kairomone-baited traps [26,37]. This is in agreement with the findings of current study with pheromone alone or pheromone + kairomone as the fan inside the new traps had significantly higher trapping percentages than the commercially available trap which operates with natural convection of air. Trapping percentages differed among the different trap designs with the presence of air flow inside the new traps. These differences in the trap catch can be attributed to trap structure, relative location of attractants (pheromone and kairomone) and number of openings in the trap designs. 

Even if onboard fans are considered impractical, the present study demonstrated that trap efficiency for *T. casteneum* can be substantially increased simply with changes to the form and design of the trap. Similar results were also observed in the traps previously tested for *T. castaneum*: Storgard trap 12%, Modified Storgard trap 20–26%, Rounded Storgard trap 12–16%, Flit Trak trap 13–26% and Fuji trap 26% [46]; dome traps 24% [26]. The different lure designs varied in the attraction response by *T. castaneum* [46]. These traps were made of cardboard with corrugations leading to a cup containing oil. Change in the direction of corrugations across cardboard layers from diagonal (in Storgard trap) to perpendicular (in modified Storgard trap) increased trapping from 11.5% to 26%. Furthermore, in the modified Storgard trap, the change in the directions of corrugations in adjacent layers would aid to maintain higher amount of pheromone inside the trap and get more insects oriented into the cup. Fuji trap (rectangular) and ‘Flit-Trak’ trap (cone shaped) both were ‘ramp and pitfall type’ and had increased trapping as 26% and 23%, respectively. While the ‘ramp’ of these two traps would have caused increased trapping, different shapes (rectangular and cone) would also have increased trapping efficiency. The rounded Storgard trap had perpendicular corrugations and big punch outs leading to a cup with pheromone. These would have contributed to move out of pheromone faster, thus decreasing its trapping than ‘Flit-Trak’ trap and Fuji trap. In addition, the round shape of the trap would also have caused decreased trapping. Trapping percentages of newly-designed traps in the current study were much higher than those obtained for in previous studies. The differences in the trap designs as well as those related to the environmental conditions and beetle-release-distance from the traps would also have accounted for these observed differences. 

More beetles were trapped when a pheromone combined with a kairomone in the same trap [47] which is in agreement with the results presented here. With the operation of fan, Trap 3 and Trap 5 had higher number of adults caught when both pheromone and kairomone were used compared to pheromone alone. In contrast, without the fan, no differences were detected in any of the newly-designed traps when used either with the pheromone alone or both pheromone and kairomone. However, the commercial trap had higher trapping percentages when both pheromone and kairomone were used compared to pheromone alone.

Attraction by insects to pheromones and kairomones is increased by odour plume created as a result of wind currents and the air movement is an important factor that increases the insect orientation to stimuli [45]. Response of *T. castaneum* to pheromone and kairomone baited traps was increased by the presence of air moving [37]. In the current study, the fan inside trap makes a strong air movement over the trap toward the beetle. It may be a reason for higher trapping percentage for all of the experiment traps compared to the commercial trap. Furthermore, in the current study Trap 3 showed higher trapping percentage than other fan-aided traps with the presence of pheromone and kairomone. In the Trap 3, the kairomone source was positioned lower than the pheromone septa and at the same vertical position. 

Corrugated paper is inexpensive, easy to fabricate, rigid and has a rough texture which many insects prefer [48]. In this study, white colour abrasive papers which are rigid and rough in texture were used for the fabrication of all the new trap designs. The higher trapping percentages demonstrated by the new traps reiterate the suitability of the type of papers as highlighted by Barak and Burkholder [48]. 

Lindgren four-unit funnel traps and Japanese beetle traps were the most effective traps for *Rhyzopertha dominica* (F.) (Coleoptera: Bostrychidae) compared to bucket trap [49]. This difference in capture of *R. dominica* were due to difference in the size of trap openings. Furthermore, these trap openings are the exhaust portions of the traps where the beetles gain unobstructed pathway into traps [49]. Similar findings were obtained in this research as the trap designs with more openings had higher trapping percentages. Trapping percentages in Trap 3 and Trap 4 each with 6 big openings had higher trapping percentage compared to the other trap designs tested.

In grain storage and food processing facilities, insects are found in places of varying air movement. For instance, air circulation is minimum under equipment, corners, in the depths of the equipment or pallets, inside warehouses. In contrast, there are facilities with ample air movement, such as sieve floor, between two rooms with different air makeup. Thus, the new traps which have varied trapping percentages with and without fan operation can be used appropriately under these different settings. Previous studies in our laboratory have shown that the number of *T. castaneum* captured increases in the presence of airflow [26]. Furthermore, as mentioned above also, the speed of air movement is variable in food storage/processing facilities [37]. The differences in the design and arrangement for air movement of the new traps may increase the potential of monitoring insects in grain storage and processing facilities but additional tests need to be designed for this. It may be interesting to test the efficiency of new traps under these conditions with air turbulence to determine if it counteracts the fan effect of the new traps. Furthermore, future studies can be designed to test the trapping efficiency of commercial traps under the said air movement conditions. 

In the current study, trapping percentages of *T. castaneum* by newly-designed traps under different conditions (availability of air flow, pheromone alone or both pheromone and kairomone) varied as 34–85% for a 3 h duration. This is comparable with the trapping of other stored-product insects by different traps for different durations of exposure (Table 3). Due to the outstanding trapping efficiencies, the new traps or their modified versions would be ideal for monitoring of *T. castaneum* in grain storage facilities but testing under different settings in warehouse or food processing facilities would be necessary.

The traps designed in the current study show increased trapping efficiency for *T. castaneum*. Use of these new traps according to correct trap density would provide a good understanding on the presence and distribution of *T. castaneum* in grain storage facilities. Further tests are required to determine the amount of air flow needed to increase trap catch, determining the duration that fans can run before batteries die. Furthermore, tests are needed in warehouses/processing facilities with their own air flows to determine if the differences seen here in simple room settings are also seen in commercial facilities. The efficacy of the new traps should also be tested with other species and pheromones. Correct understanding on the response of stored-products beetle species to the newly-developed traps would enhance the possibility of using of them as monitoring tools in IPM programs targeting better protection of stored and processed food in different facilities.

## 5. Conclusions

The current study demonstrated that changes in the design of traps and placement of pheromone and kairomone within traps could markedly increase the capture efficiency of monitoring traps for *T. castaneum*. Factors associated with improved performance included differences in shape, rougher entry surfaces, covered trap tops, placement of pheromone and kairomone within the trap, and forced ventilation from an internal fan. Testing the newly-designed traps for trapping of other stored-product insects would further enhance the potential use of these traps in stored-product pest management.

## Figures and Tables

**Table 1:** The list of materials used for trap development.

Material	Trap 1	Trap 2	Trap 3	Trap 4	Trap 5	Cost of Materials
A small motor	1	1	1	1	1	0.162
1.5 V battery	1	1	1	1	1	0.189
Abrasive paper	1	1	1	1	1	0.405
Exhaust fan	1	1	1	1	1	0.108
Electric wire	20 cm	20 cm	20 cm	20 cm	20 cm	0.021
Plastic vial	-	3	3	3	1	0.216
Nylon net	-	100 cm	-	-	150 cm	0.108
Plastic funnel	1	-	-	-	-	0.108

**Table 2:** Percentage (mean ± SE) *T. castaneum* adults trapped in different trap designs with either pheromone alone or pheromone + kairomones operated either with or without fan (4 replicates, Number of adults released per replicate 200, duration of exposure 3 h).

Trap Design	% Trapped (Mean ± SE) ^1^			
	No Fan		Fan	
	Pheromone	Pheromone + Kairomones	Pheromone	Pheromone + Kairomones
Commercial Trap	12.6 ± 1.9 eB	23.6 ± 0.6 eA	na	na
Trap 1	34.4 ± 0.9 dA	36.0 ± 0.9 dA	36.1 ± 0.8 eA	37.0 ± 3.1 eA
Trap 2	33.75 ± 2.3 dB	33.88 ± 2.0 dB	47.5 ± 1.9 dA	49.25 ± 1.5 dA
Trap 3	60.0 ± 1.4 aC	65.75 ± 1.9 aC	75.0 ± 1.3 aB	85.13 ± 1.5 aA
Trap 4	49.75 ± 1.0 bB	50.5 ± 0.7 bB	58.0 ± 0.4 cA	59.13 ± 1.3 cA
Trap 5	42.0 ± 0.4 cC	42.5 ± 1.3 cC	65.38 ± 1.2 bB	75.0 ± 1.7 bA

^1^ Means followed by the same lowercase letter in a column and the same uppercase letter in a row are not significantly different according to Tukey’s test (*p* < 0.05) following ANOVA.

**Table 3:** Trapping efficiencies of different traps for stored-product insect species.

Trap	Species	Trapped (%)	Duration of Exposure (days)	Reference
Storgard trap	*Oryzaephilus surinamensis*	64	0.7	[42]
Storgard trap	*Tribolium confusum*	95	0.7	[42]
Pitfall traps	*Lasioderma serricorne*	30	2	[50]
Sticky traps	*Plodia interpunctella*	20	3	[50]
Dome trap	*T. confusum*	2	10	[25]

## Supplementary Materials

The following are available online at https://www.mdpi.com/2075-4450/11/11/778/s1, Trap Descriptions. Figure S1: Different views of trap 1, Figure S2: Different views of trap 2, Figure S3: Different views of trap 3, Figure S4: Different views of trap 4, Figure S5: Different views of trap 5.
