# Supplementary material for: Changes in Shape, Texture and Airflow Improve Efficiency of Monitoring Traps for Tribolium castaneum (Coleoptera: Tenebrionidae)
Source: Insects. 2020 Nov 10;11(11):778. doi: 10.3390/insects11110778 (PMC7696696; doi:10.3390/insects11110778)
Supplement: Supplementary file 1 [file insects-11-00778-s001.pdf]

Trap Descriptions

Trap 1: Trap is hexagonal in shape. Apex of each side joins the horizontal top cover with an opening. The centre of horizontal top cover contains three circular openings. Exhaust fan is fixed to the centre of bottom plate. A circular plastic cup (having kairomones) and a circular plastic groove (to collect insects) are placed at the centre and the periphery, respectively above the fan separated by a nylon mesh cover. The six openings at the apex of six sides vertically align with the circular plastic groove inside the trap. Furthermore, the three central openings on the horizontal top cover align with the circular plastic cup inside. Three pheromone-containing grips housed above the mesh (and held in place attached to circular plastic groove) are placed at the same level with the upper margin of kairomones cup. *Tribolium castaneum* adults move into the trap through three circular openings at the top centre position are collected into the circular cup having the kairomone. The insects move in through the opening at the apex at each side are collected in the circular plastic groove. Alternatively, the insects enter the trap through the three central openings are collected in the kairomone-containing cup (Figure S1).

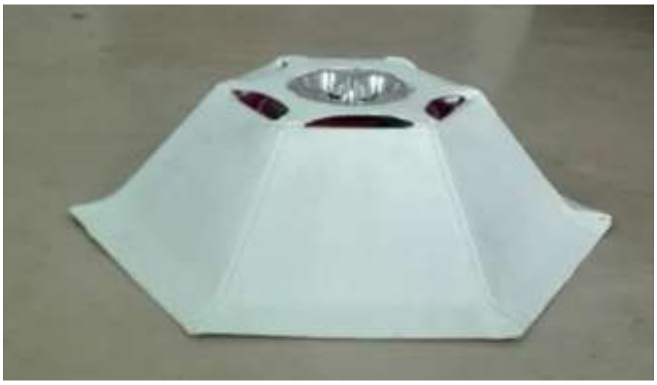

Side view of the trap

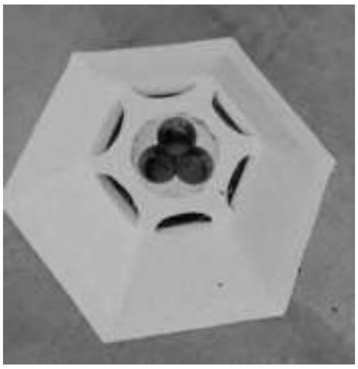

Top view of the trap outside

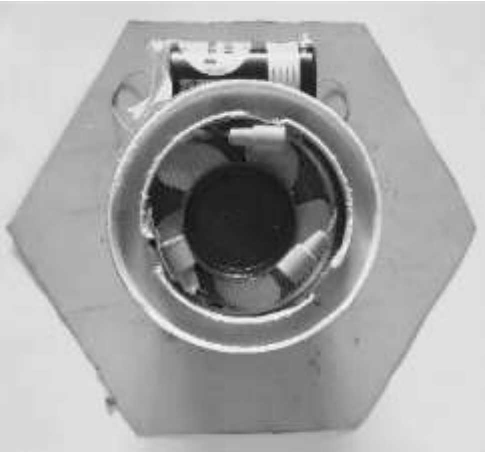

Top view of bottom plate

Figure S1: Different views of trap 1.

Trap 2: Trap is triangular in shape. One opening on each side at the bottom. Top half of each opening is covered by the net material whereas the bottom half is left bare open. Exhaust fan is located inside the trap on the bottom plate to be at the centre. Three pheromone-containing grips and three kairomone-containing circular cups are placed below the fan. Each pheromone grip is attached by metal wire to the single vertex. The kairomones cups are placed in the depressions inside each side of the trap. *Tribolium castaneum* adults move in through the three triangular openings, and are collected into the triangular hole inside the trap having the kairomone (Figure S2).

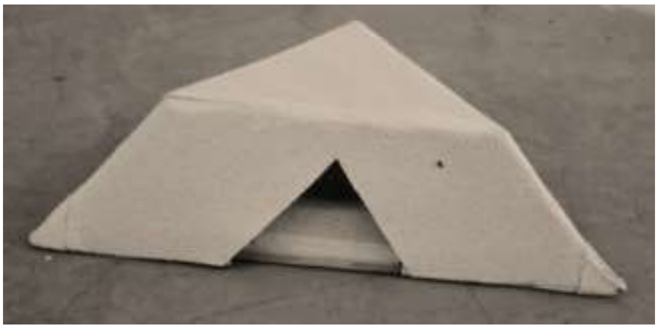

Side view of the trap

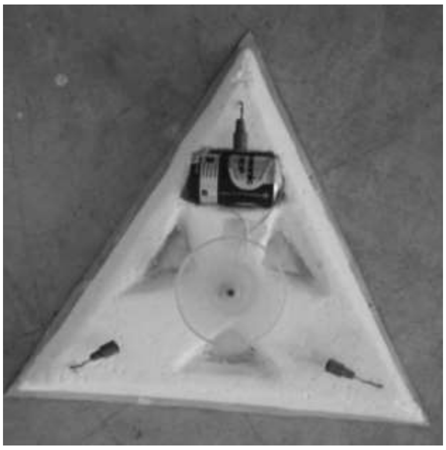

Inside view of top cover

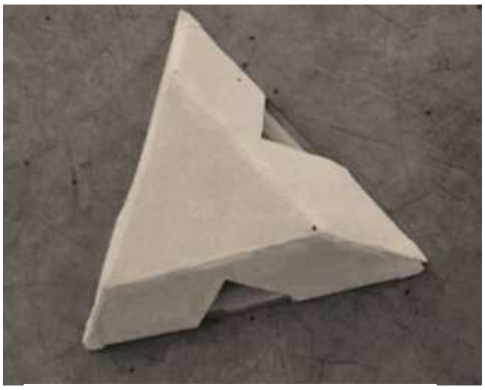

Top view of trap outside

Figure S2: Different views of trap 2.

Trap 3: Trap is triangular in shape. Each side contains two vent holes covered by netting material; in total six vent holes. Each side has a horizontally-placed opening (for the entry of insects) to which internally a pheromone-containing rubber grips is attached by a metal wire; in total three pheromone grips. The bottom plate of the trap internally has an exhaust fan located to be at the centre. Three kairomone-containing cups are placed on the bottom plate inside the trap to be below the fan and directly underneath the pheromone-containing rubber grips (located above the fan). *Tribolium castaneum* adults are collected in to the three cups having the kairomone (Figure S3).

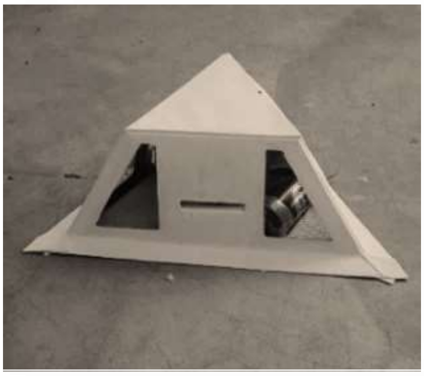

Side view of the trap

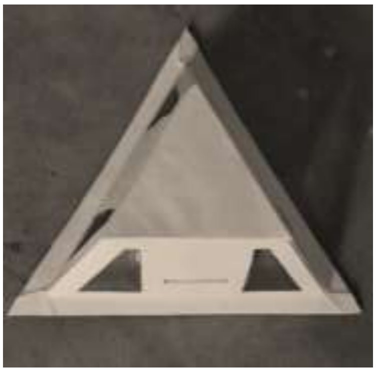

Top view of trap outside

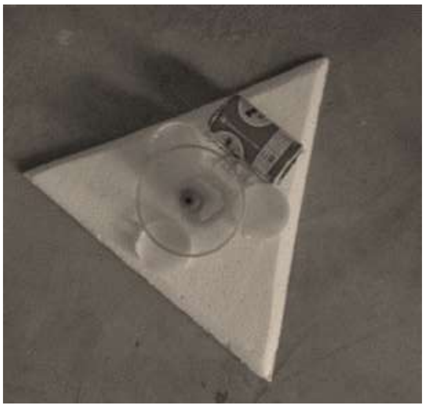

Bottom portion of trap inside

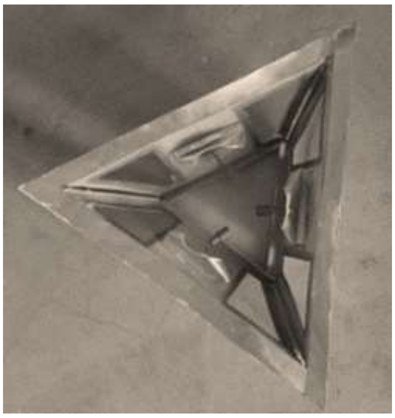

Inside view of top cover

Figure S3: Different views of trap 3.

Trap 4: Trap is triangular in shape. Each side contains two vent holes covered by netting material; in total six vent holes. Each side of the trap has a horizontal opening (for the entry of insects). Internally to each 'horizontal opening', a kairomones-containing cup (above) and a second cup to collect the insects entering the trap are located. The bottom plate of the trap internally has an exhaust fan located to be at the centre. Three pheromone-containing rubber grips are attached to the bottom plate each by a metal wire; in total three pheromone grips in the trap. The kairomone-containing cups are attached inside the top cover to be above the fan and directly on the same line with the pheromone-containing rubber grips (located below the fan) (Figure S4).

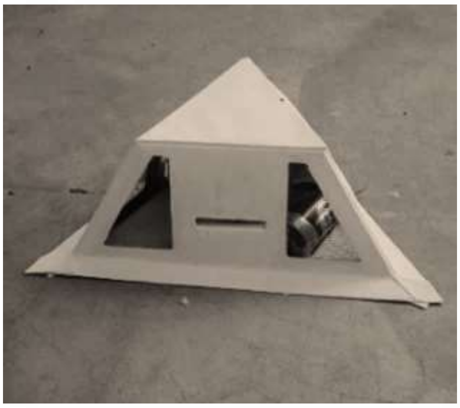

Side view of the trap

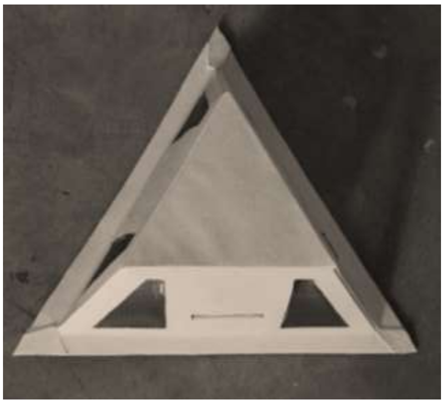

Top view of trap outside

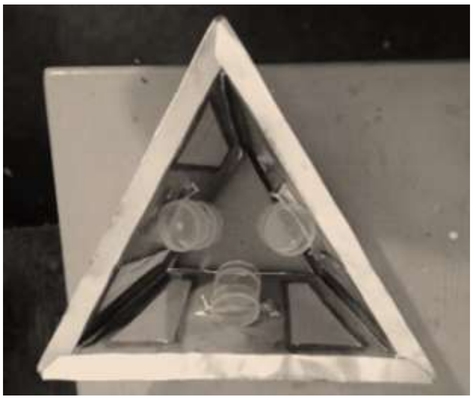

Inside view of top cover

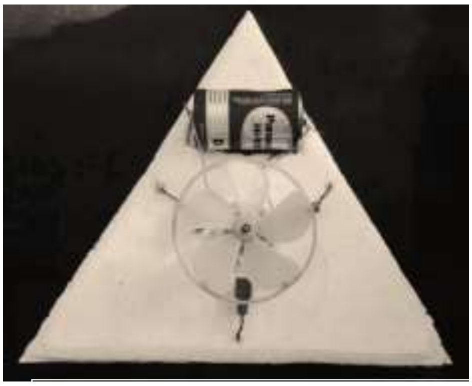

Bottom portion of trap inside

Figure S4: Different views of trap 4.

Trap 5: Trap is square shaped. Each of the two opposite sides of the trap have one centered-opening at the bottom covered by netting material (meant for the release of pheromone and kairomone). The other two opposite sides have two small bare openings (through which *T. castaneum* beetles enter) each locates approximately at ¼ length from the edge. The inside centre position of the bottom plate of the trap houses the exhaust fan. The inside surface of the top cover of the trap contains three pheromone-containing grips (attached by metal wires) and two-kairomone containing cups (attached to the top surface leaving approximately 0.5 cm distance from the cup opening) to be on either side of the fan housed at the centre of the bottom plate. The pheromone grips and kairomone cups are placed to be at the same level as the fan when the top and bottom portions of the trap are united (Figure S5). *Tribolium castaneum* adults are collected into the two kairomone cups and two rectangular alleys inside the trap.

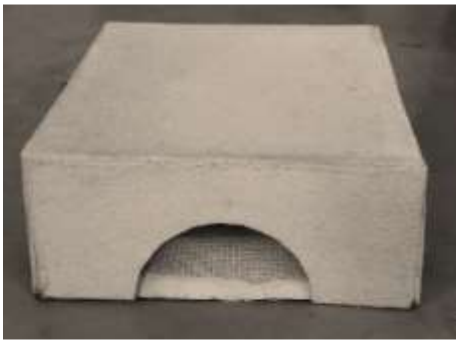

Side view of the trap

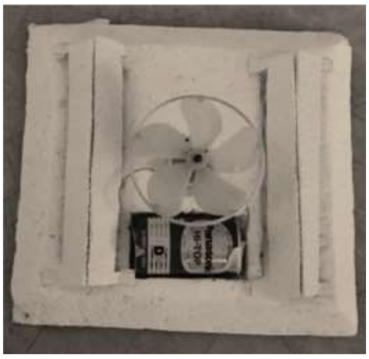

Bottom portion of the trap inside

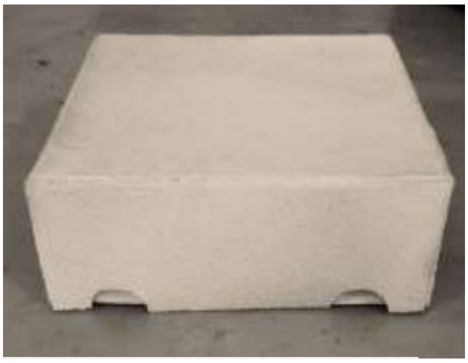

Side view of the trap

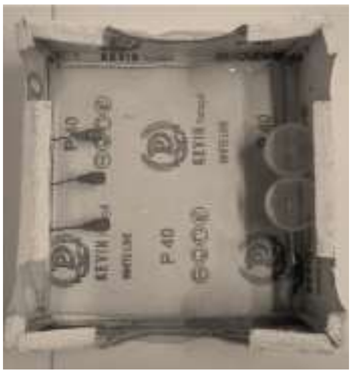

Inside view of top cover

Figure S5: Different views of trap 5.
